# Aggressive NK Cell Leukemia: Current State of the Art

**DOI:** 10.3390/cancers12102900

**Published:** 2020-10-09

**Authors:** Siba El Hussein, L. Jeffrey Medeiros, Joseph D. Khoury

**Affiliations:** Department of Hematopathology, The University of Texas MD Anderson Cancer Center, Houston, TX 77030, USA; sel2@mdanderson.org (S.E.H.); ljmedeiros@mdanderson.org (L.J.M.)

**Keywords:** leukemia, NK cell, molecular, immunohistochemistry, biomarker

## Abstract

**Simple Summary:**

Aggressive natural killer cell leukemia (ANKL) is a rare, lethal disease that presents many diagnostic and therapeutic challenges. Recent studies have shed new light on the salient features of its molecular pathogenesis and provided further insight into the clinicopathologic spectrum of this disease. This review presents a state-of-the-art overview of ANKL, spanning its historical evolution as a distinct entity, pathobiology, and potential therapeutic vulnerabilities.

**Abstract:**

Aggressive natural killer (NK) cell leukemia (ANKL) is a rare disease with a grave prognosis. Patients commonly present acutely with fever, constitutional symptoms, hepatosplenomegaly, and often disseminated intravascular coagulation or hemophagocytic syndrome. This acute clinical presentation and the variable pathologic and immunophenotypic features of ANKL overlap with other diagnostic entities, making it challenging to establish a timely and accurate diagnosis of ANKL. Since its original recognition in 1986, substantial progress in understanding this disease using traditional pathologic approaches has improved diagnostic accuracy. This progress, in turn, has facilitated the performance of recent high-throughput studies that have yielded insights into pathogenesis. Molecular abnormalities that occur in ANKL can be divided into three major groups: JAK/STAT pathway activation, epigenetic dysregulation, and impairment of *TP53* and DNA repair. These high-throughput data also have provided potential therapeutic targets that promise to improve therapy and outcomes for patients with ANKL. In this review, we provide a historical context of the conception and evolution of ANKL as a disease entity, we highlight advances in diagnostic criteria to recognize this disease, and we review recent understanding of pathogenesis as well as biomarker discoveries that are providing groundwork for innovative therapies.

## 1. Introduction

Aggressive natural killer (NK) cell leukemia (ANKL) is a rare, fulminant disease with a dismal prognosis that presents many diagnostic challenges. Since its original description in the 1980s, the diagnostic approach to ANKL has evolved slowly, in large part due to disease rarity and the complexity of clinical and pathologic presentations. An additional major challenge has been a lack of a specific immunophenotypic or molecular signature characteristic of ANKL. Most early studies attempted to provide a systematic approach to the diagnosis ANKL by relying on clinical and morphologic correlation, coupled with ancillary tools such as flow cytometry and conventional karyotyping. Although robust, these tools did not uncover specific biomarkers, partly because traditional approaches lack the versatility inherent in next-generation sequencing, particularly in the context of an NK cell neoplasm.

In recent years, high-throughput genomic analyses at the DNA and RNA levels has contributed greatly to our understanding of ANKL and provided novel grounds for potential targeted therapies. This review is intended to provide an overview of ANKL, as defined in the current World Health Organization (WHO) classification [1], spanning its historic origin, clinical and laboratory manifestations, biology, and novel therapeutic approaches, with emphasis on advances in our understanding of this rare malignancy in light of recent advances. This review is based on a review of the literature using the term “aggressive NK cell leukemia”.

## 2. Historical Overview

The recognition of ANKL as a separate entity was presaged by the disease group referred to as NK-type lymphoproliferative disease of granular lymphocytes (NK-LDGL). Entailing abnormal proliferation of large granular lymphocytes (LGLs) expressing a characteristic NK cell immunophenotype (CD3−/CD56+/CD16+), NK-LGDL was understood to encompass heterogeneous clinical manifestations ranging from indolent, asymptomatic conditions to aggressive, fulminant diseases. As a body of informative work underscoring some characteristics of NK-LGDL emerged [2,3,4,5], gradual attention was turned to separating indolent forms from those with an acute clinical course. Thus eventually emerged from NK-LDGL the entities of NK-LGL, chronic lymphoproliferative disorder of NK cells (CLPD-NK), and ANKL.

The term ANKL was first mentioned in a publication by Fernandez et al. in 1986 [6]. The authors described a case of a 70-year-old man with multiple episodes of intestinal perforation of unknown cause, splenomegaly, and bone marrow infiltration by cells described as being morphologically similar to large granular lymphocytes (LGLs), but lacking T cell markers. The patient had a rapidly progressive clinical course and died within two months of hospital admission. At autopsy, he had extensive involvement of the bone marrow, spleen, lymph nodes, and jejunum by LGL-like cells. Conventional cytogenetic analysis revealed clonal aberrations, leading the authors to conclude that the disease was neoplastic. The authors also postulated that this disease represented an aggressive form of NK cell leukemia.

In 1988, Imamura et al. reported two cases of NK cell leukemia/lymphoma that lacked clonal rearrangements of the T-cell receptor (*TRB*, *TRG)* and immunoglobulin genes [7]. The authors noted that the neoplastic cells in these two cases expressed CD56 (NKH-1/Leu-19) and lacked CD57 (HNK-1/Leu-7). Two years later, the same authors compared four cases of ANKL to seven other similar cases reported in the literature [6,8,9,10,11]. Imamura and colleagues concluded that this disease emanates from “a third lineage of lymphoid cells” and used the term “aggressive natural killer cell leukemia/lymphoma” [12].

ANKL was subsequently reported to arise in two patients with “lethal midline granuloma”, currently known as extra-nodal NK/T cell lymphoma, nasal type (ENKTL), raising the possibility that ANKL disease could represent a terminal event in the evolution of ENKTL [13]. This report was followed by another, which described four cases of ANKL with dissemination to various organs in a manner akin to lymphomas [14]. These reports raised controversy regarding the relationship between ANKL and ENKTL, which arguably remains largely unresolved [15,16].

In 1994, the Revised European–American classification of lymphoid neoplasms recognized ANKL as an entity separate from ENKTL, and such separation was adopted in the WHO classification system [17,18].

## 3. Clinical Features

ANKL patients have a median age of 40 years, and there is no gender predilection. The disease has a higher incidence in Asian populations, but ANKL can also arise in other groups [19]. The most common presenting signs and symptoms include fever, B-symptoms, hepatosplenomegaly, lymphadenopathy, disseminated intravascular coagulopathy, and hemophagocytosis. Epstein-Barr virus (EBV) infection has been observed in a subset of ANKL cases. Despite intensive chemotherapy, patients with ANKL have a very poor prognosis, with a median survival of less than two months [20,21]. Several prognostic factors have been proposed, including patient age, serum lactate dehydrogenase level, and serum total bilirubin level [22], but these factors have not been validated [23]. The clinicopathologic features of ANKL have been elucidated in several case series [19,20,21,24,25,26,27].

## 4. Morphologic Features

**Figure 1:**
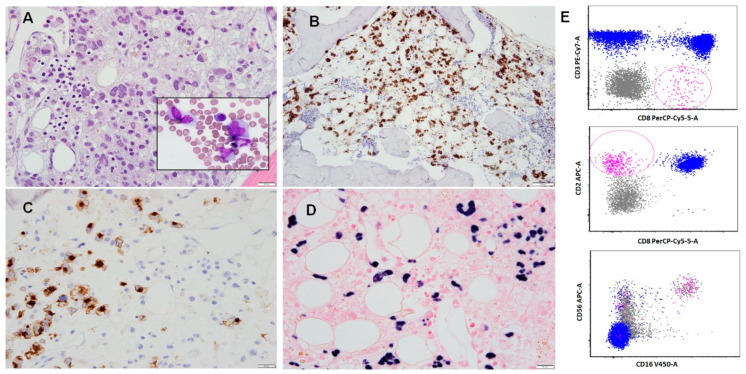
Aggressive natural killer (NK) cell leukemia involving bone marrow. (**A**) Trephine biopsy showing an extensive infiltrate of neoplastic lymphocytes in interstitial and less pronounced sinusoidal infiltration patterns. (**B**) CD2 immunohistochemistry, positive in neoplastic cells. (**C**) Perforin A immunohistochemistry, positive in neoplastic cells. (**D**) Epstein–Barr virus encoded small RNA (EBER1/2) expression in neoplastic cells. (**E**) Flow cytometry immunophenotyping performed on an aspirate sample demonstrated an aberrant population of lymphocytes (pink highlight), positive for CD2, CD8, CD16, and CD56. These cells were negative for CD3 and CD5. In addition, they were negative for CD4, CD57, T cell receptor (TCR)-alpha/beta, and TCR-gamma/delta.

Histologically, bone marrow involvement by ANKL can be prominent or subtle, with two main patterns of infiltration: interstitial and sinusoidal [19]. The neoplastic cells are generally medium-sized, possess a moderate amount of cytoplasm, and exhibit highly irregular nuclei with condensed chromatin and conspicuous nucleoli. Apoptosis and focal necrosis are common findings; however, geographic necrosis is typically not observed in ANKL, unlike ENKTL [19] (Figure 1A). Microscopic examination of peripheral blood and bone marrow smear preparations usually demonstrate neoplastic cells that are intermediate to large in size, with moderate amounts of basophilic agranular cytoplasm often containing punched-out vacuoles. The neoplastic cells typically have highly irregular nuclear contours and an open chromatin pattern with prominent nucleoli.

## 5. Immunophenotypic Features

When assessing normal NK cells by multiparameter/multicolor flow cytometry immunophenotyping, 90% were CD56^dim^/CD16^bright^, and 10% were CD56^bright^/CD16^dim^ [27]. It is thought that the CD56^dim^/CD16^bright^ group represents a more mature stage of NK cell differentiation, but prior to mature NK cells with expression of CD57 and killer immunoglobulin-like receptors (KIRs). The CD56^bright^/CD16^dim^ group represents an earlier stage of differentiation [19,28,29]. Following this rationale, NK cell neoplasms can be divided into two broad groups. The first group is CD56^bright/positive^/CD16^dim/negative^, suggesting failure of differentiation [30]. This group, which encompasses ANKL and ENKTL, retains a cytokine-secreting function that underlies the cytokine storm that occurs characteristically in patients with these two diseases [30]. The second group is CD56^dim/negative^/CD16^bright/positive^, encompassing chronic lymphoproliferative disorder of NK cells (CLPD-NK), and characterized by neoplastic cells with a cytotoxic function [30,31] (Table 1).

The main flow cytometry features of ANKL are prominent forward scatter (FSC), denoting an increased cell size when compared to non-neoplastic background lymphocytes, and expression of CD56 and CD94 [19]. CD56 is a neural cell adhesion molecule that assists in infiltration and invasion. CD94 is a molecule that forms heterodimers with either NKG2A (CD159a) or NKG2C (CD159c). The lectin-like CD94/NKG2A receptor is inhibitory and recognizes the human leukocyte antigen (HLA) class Ib molecule HLA-E as its predominant ligand [32,33], while the CD94/NKG2C is activating/triggering [34,35]. While Zambello et al. have demonstrated that in the vast majority of NK-LGDL cases (16/18; 88.9%) CD94 is associated with NKG2A [5], to our knowledge the functional associations of CD94 in ANKL has not been characterized yet. ANKL cells appear to consistently express CD2, in addition to cytoplasmic CD3 (epsilon chain), CD16, and cytotoxic molecules, such as granzyme B, TIA1, and perforin A. Expression of CD7 or CD8 is heterogeneous, whereas CD3 (surface), CD4, CD5, CD57, and T cell receptors (TCR) are usually absent in ANKL [36]. The KIR (killer cell immunoglobulin-like receptor) proteins (CD158a/b/e), known to be present only in peripheral blood CD56^dim^ NK cells, are mostly negative in ANKL [19,36]. In practical terms, neoplastic NK cell populations can be separated from lymphocytes using a CD45/SSC gating strategy. In the case of neoplastic NK cells overlapping with lymphocytes, using a CD56/CD3 gating strategy is helpful to identify the aberrant cells [36] (Figure 1B–E and Table 2).

One challenge to establishing an early diagnosis of ANKL is the common presence of only a small number of neoplastic NK cells in the bone marrow [19]. In one study of ANKL, the proportion of abnormal NK cells in the bone marrow was reported to be <5% in half of the patients [27]. Flow cytometry immunophenotypic analysis is particularly useful in the early detection of ANKL, usually with a high specificity [27]. As ANKL progresses, the number of neoplastic cells in the bone marrow increases. In one study, the rate of detection of aberrant NK cell populations increased from 86.8% to 97.4% when second and third bone marrow samples were assessed as the disease progressed [27]. Thus, it is advisable to obtain several specimens from the blood, bone marrow, or other tissue sites and to conduct consecutive flow cytometry analyses when positivity is not detected and the patient is suspected clinically to have a neoplasm such as ANKL [27] (Figure 1E).

Homing of neoplastic NK cells to body cavities has been outlined recently in NK cell neoplasms, including ANKL, creating a differential diagnosis with primary effusion lymphoma [37,38,39,40]. This differential diagnosis can be addressed successfully by using flow cytometry immunophenotyping when morphologic examination is impeded or is of limited value [36].

## 6. The Role of Epstein–Barr Virus

The first cases of ANKL reported by Fernandez et al. and Imamura et al. were EBV-negative or EBV was not assessed [6,12]. Kawa-Ha et al., in 1989, were the first to highlight that neoplastic NK cells in the “aggressive” and “chronic” forms of “lymphoproliferative disease of granular lymphocytes” (currently, ANKL and T cell LGL, respectively) could carry EBV DNA, thus implicating EBV in the pathogenesis of these diseases [41]. EBV-positive cases are positive for EBV-encoded RNA (EBER) and are negative for latent membrane protein type 1 (LMP-1) in some reports, suggesting a type I latency pattern of infection [24,25]. This latency pattern is believed to provide an advantage to neoplastic NK cells by enabling them to evade host virus-specific cytotoxic T cell activity [42,43].

Although ANKL has an established association with EBV infection, EBV-negative ANKL is also widely recognized [19,20,21,44,45,46,47,48]. In contrast with EBV-positive ANKL, EBV-negative cases occur more frequently in older patients and arise equally in Asian and non-Asian populations [44,45]. No morphologic or immunophenotypic differences have been shown in EBV-positive versus EBV-negative ANKL [45]. Although some reports have suggested a more indolent behavior of EBV-negative ANKL [46,49,50], recent articles have shown that these cases are associated with an aggressive clinical behavior, similar to EBV-positive cases [44,45].

The absence of EBV in a subset of ANKL cases can potentially lead to a delayed diagnosis, with adverse clinical consequences. When evaluating a potential case of ANKL, one should not use EBV-negative status as a criterion to exclude the diagnosis. Further investigation of these neoplasms is needed to unmask NK cell lineage. Performing immunohistochemical (IHC) analysis can be helpful because CD56 and cytotoxic molecules are reported to be positive in most ANKL cases [44,45].

## 7. Cytogenetic Features

Conventional cytogenetic abnormalities in ANKL have been reported in few studies, and these abnormalities commonly include del (6) (q21q25) and del (11q) [24,25,51]. Another study found an association between ANKL and chromosome 7 abnormalities, and also reported chromosome 6q deletion associated with ENKTL [52]. More recently, array-based comparative genomic hybridization (aCGH) analysis in a cohort of patients with ANKL and ENKTL showed that gains of 1q23.1–q23.2 and 1q31.3–q44, as well as losses of 7p15.1–q22.3 and 17p13.1, are more frequent in ANKL than ENKTL [53]. In contrast, cases of ENKTL more frequently had gains of 2q and losses of 6q16.1–q27, 11q22.3–q23.3, 5p14.1–p14.3, 5q34–q35.3, 1p36.23–p36.33, 2p16.1–p16.3, 4q12, and 4q31.3–q32.1 [53]. Others have reported that loss of 6q16.1–q27 is a common finding in NK cell malignancies [54], detected in both ANKL [24,25,51] and ENKTL, but more frequently in ENKTL [53].

## 8. Molecular Pathogenesis and Genomic Landscape

Abundant data are available with regards to genetic pathways involved in the pathogenesis of ENKTL. These abnormalities include mutations in the JAK/STAT [55,56,57,58,59], AKT [59], and NF-κB [59] signaling pathways. Additional abnormalities include recurrent chromosomal aberrations in ENKTL including a 6q21 deletion [60] (silencing the tumor suppressors PRDM1 and FOXO310), and mutations in the RNA helicase gene DDX3X [61]. However, apart from copy number aberration analyses [24,25,53], a deeper understanding of the genetic alterations in ANKL was missing until recently. The advent of next generation sequencing methods has provided an unprecedented impetus for understanding the molecular pathogenesis of ANKL. Three recent studies [19,62,63] provided a comprehensive genetic analysis of ANKL via next generation sequencing (Table 3). While the findings in these studies show some differences that are likely the result of differing methodologies, they share many common threads that provide insight into the molecular landscape of ANKL. Major genetic findings in these studies are discussed below:

### 8.1. JAK/STAT Signaling Pathway

Four JAKs (JAK1, JAK2, JAK3, TYK2) and seven STATs (STAT1, STAT2, STAT3, STAT4, STAT5a, STAT5b, STAT6) are used by more than 50 cytokines and growth factors [64]. Extracellular binding of cytokines or growth factors with their corresponding trans-membrane receptors induce conformational changes in receptor-bound JAK proteins, creating a distance between their kinase domains and inhibitory pseudo-kinase domains [65]. Trans-activated JAK proteins subsequently phosphorylate STAT proteins, resulting in dimerization, nuclear translocation, and direct DNA binding [65]. STATs disperse throughout the genome and regulate transcription of both protein-coding and non-coding genes [66]. In addition, all STATs recognize the same DNA sequence, known as the GAS motif [64]. However, STATs may antagonize the action of one another by competing for the binding of the same genomic site [64]. For example, in T cells, dendritic cells, and cancer cell lines, *STAT3*-driven IL-17 transcription is blocked by *STAT5* [67,68,69].

Dufva et al. analyzed 14 patients of ANKL using whole-exome sequencing [62]. They showed frequent genetic mutations in the JAK/STAT (21% of cases exhibited *STAT3* mutations) and RAS-MAPK signaling pathways [62]. Similarly, Huang et al. analyzed eight patients with ANKL by whole-genome sequencing (WGS) and 29 ANKL (including the eight patients analyzed by WGS) patients by targeted sequencing [63]. They noted that mutations in molecules of the JAK/STAT system, namely, *STAT3*, *STAT5B*, *STAT5A*, *JAK2*, *JAK3*, *STAT6*, *SOCS1*, *SOCS3*, and *PTPN11*, were seen in 48% of patients, with 17% of the cases harboring *STAT3* mutations [63]. El Hussein et al. analyzed six ANKL patients, finding mutations in *JAK1* in one patient (who also harbored a mutation in STAT3), *JAK3* in one patient, and *STAT3* in three patients [19]. In general, mutations in the JAK/STAT pathway were mutually exclusive.

Huang et al. [63] and Dufva et al. [62] found that most of the *STAT3* and *STAT5B* mutations were localized to exons 20 and 21 encoding the Src homology 2 (SH2) domain, which mediates STAT protein dimerization [63]. This domain also constitutes the hotspot containing activating mutations in NKTCL [55,61]. Other JAK/STAT-related mutations reported were 9p copy number gain-containing JAK2 and a point mutation in the protein tyrosine phosphatase (PTP) PTPRK, as well as mutations in PTPN4 and PTPN23 [62]. Of these molecules, PTPRK is a tumor suppressor shown to negatively regulate STAT3, and is commonly deleted in NKTCL.

Huang et al. suggested that EBV-encoded small RNAs (EBERs) that are highly expressed in some cases of ANKL induce the release of massive amounts of IL-10, a well-known upstream activator of the JAK/STAT pathway [70]. This stimulation in turn stimulates *STAT3* phosphorylation, leading to downstream MYC activation [63]. Importantly, Huang and colleagues also observed that JAK/STAT-mutated and -unmutated leukemic NK cells showed a similar expression pattern in *MYC*-driven programs [63]. This finding suggests that *STAT3* signaling can be activated by over-production of IL-10 or through other unknown mechanisms, independent of genetic mutations [63]. This observation has been supported by immunohistochemical analysis, which has shown phosphorylated STAT3 (p-STAT3; indicative of activation) is significantly higher in neoplastic cells residing in the bone marrow of both JAK/STAT wild-type and mutated ANKL cases as compared with controls [63].

The high frequency of mutations involving the JAK/STAT signaling pathway prompted Huang et al. to analyze the plasma levels of inflammatory cytokines in patients with ANKL [63]. They found significantly high serum levels of IL-10 in ANKL patients [63]. The authors concluded that IL-10 plays a major role in upstream activation of the JAK/STAT pathway in ANKL, leading to increased expression of *MYC* [63]. The authors subsequently proposed an IL-10–STAT3–MYC transcription regulation model involved in the pathogenesis of ANKL [63]. On another note, IL-10 treatment of cell lines in the same study preferentially stimulated JAK/STAT wild-type cells, but not the *STAT3* Y640F-mutant ANKL cells, causing an increase pathway activity and cellular proliferation [63]. This result suggests that IL-10 upregulation plays a more prominent role in JAK/STAT unmutated ANKL patients [63].

### 8.2. Epigenetic Dysregulation

Half of the cases in the Dufva et al. study harbored mutations in epigenetic regulatory molecules such as *TET2* and *CREBBP*, and four (28%) patients had mutations in the RNA helicase *DDX3X* [62]. Huang and colleagues also identified mutations in epigenetic modification-related genes, including *TET2* (28%), *CREBBP* (21%), and *MLL2* (21%) [63]. In contrast, *DDX3X* and *BOCR*, commonly found in ENKTL [61,71], were less frequently mutated in ANKL [63]. Our group also has found mutations in *TET2*, *CREBBP*, and *GFI1* in ANKL cases [19].

### 8.3. TP53 Alterations and DNA Repair

Huang et al. identified *TP53* mutations in 34% of cases of ANKL [63], whereas Dufva and colleagues identified *TP53* mutations in 1 out of 14 patients [62]. In our study, we found *TP53* mutations in three of six patients, and demonstrated uniform absence of p53 protein expression in four of eight ANKL cases assessed by immunohistochemistry [19]. This finding is in keeping with observations in an earlier aCGH study by Nakashima et al., who reported 17p13.1 losses more frequently in ANKL than ENKTL cases [53].

Dufva and colleagues noted the lack of a DNA double-strand break repair-associated signature in ANKL cases when compared with ENKTL, CLPD-NK, and T-LGL, suggesting a divergence in underlying mutational processes between ANKL and ENKTL [62]. However, no mutations specific to ANKL were uncovered in any of the recent studies [62,63]. Moreover, several genes that have been identified in ENKTL cases, such as *DDX3X* [61], *STAT3* [55,61], *BCOR* [71], and *KMT2D* [61], were also identified in ANKL [62,63]. From these observations, we suggest that one can infer that the pathogenesis of ANKL and NKTCL is closely related. Nevertheless, mutations in genes such as *DDX3X* and *TP53* were reported to contribute to a poorer prognosis in ENKTL [61], whereas the distribution of these same mutations was not homogeneous in recent studies of ANKL (Table 1). This observation suggests that mutations *DDX3X* and *TP53* play a minor role in ANKL and refutes the hypothesis that ANKL evolves from ENKTL. It is also worth mentioning that although a connection between EBV status and mutational signature was suggested in past studies [44], this theory appears to have weakened because the investigation of additional ANKL cases have been frequently EBV-negative. Several gene mutations identified in EBV-positive cases also have been identified in EBV-negative cases, such as STAT3 [45], TP53 [44], TET2 [44], and DDX3X [44]. Therefore, although EBV plays an undeniable role in the pathogenesis of a subset of cases of ANKL, other epigenetic factors may exert similar alterations on the genomic level, resulting in ANKL independent of EBV.

## 9. Examples of Clinicogenomic Data Integration

An example of genomic and clinical data integration is illustrated by the work of Tang et al. [22]. These authors identified 29 cases of classic ANKL with a fulminant presentation and eight cases of ANKL with “subacute clinical course”. The latter group was defined as patients who manifested infectious mononucleosis-like symptoms for more than 90 days, before full-blown manifestations of ANKL developed. This subacute group is important to recognize because it can be mistaken it for a self-limited infectious disease, likely delaying intervention with adverse clinical consequences [22]. By applying ultra-high multiplex PCR technology, they identified mutations in *TP53* (most commonly), genes in the *JAK/STAT RAS-MAPK* signal transduction systems, in the transcription factors *NF-κB1* and *NF-κB1A*, and in the epigenetic regulatory molecules *TET2* and *CREBBP* [22]. *TP53* was mutated significantly less often in “subacute” ANKL (none were detected) in comparison to fulminant ANKL [22]. However, gene mutations in the JAK/STAT pathway were similar between the two groups, suggesting that the key driving mechanisms are similar between the classic and subacute variants of ANKL [22].

El Hussein et al. published an integrative genomic and immunophenotypic landscape study of 12 cases of ANKL, of which six cases had available next generation sequencing data [19]. The authors also identified mutations in the JAK/STAT (*JAK1*, *JAK3)* and RAS/MAPK pathways (*KRAS*), as well as in epigenetic modifiers (*TET2*, *CREBBP*, and *GFI1*), cell cycle regulation, and DNA damage repair (*TP53*, *ASXL1*, *ASXL2,* and *BRINP3*) and mRNA splicing factors (*PRPF40B*) (Table 3). In addition, the authors coupled these genomic findings with immunophenotypic analysis by immunohistochemistry, showing frequent loss of p53 expression, in addition to overexpression of BCL-2 and MYC in ANKL cases. To date, this study includes the largest number of cases at a single institution analyzed by next-generation sequencing performed from a clinical perspective, as other studies, although highly valuable, were performed more from translational and investigational perspectives.

## 10. Immune Checkpoint Status

Gao et al. reported CD274 (PD-L1) overexpression in two of three ANKL cases [44]. Furthermore, our group has shown PD-L1 expression in two of eight ANKL cases [19]. In a small group of patients with ANKL and ENKTL, *JAK2* gain was associated with gains of the neighboring immune evasion-associated *CD274* (PD-L1) and *CD273* (PD-L2) genes [62]. In addition, sensitivity of ENKTL to PD-1 inhibition was recently outlined [72]. Taking all of these data into account, PD-1 inhibition represents a promising approach for ANKL treatment. This may be particularly true in EBV-positive cases of ANKL, as EBV infection plays a role in PD-L1 stimulation [73].

## 11. Treatment of ANKL

### 11.1. Chemotherapy in ANKL

No consensus chemotherapeutic regimen has been established to manage patients with ANKL as the rarity of this disease has precluded prospective clinical trials [23]. Current knowledge of therapy is based on small clinical studies focused on chemotherapeutic approaches and clinical outcomes. These studies have shown a role for anthracycline-containing chemotherapy regimens as ANKL patients have shown a complete response [20]. Subsequently, a role for L-asparaginase in treating ANKL was recognized. Treating ANKL cell lines with L-asparaginase results in apoptosis [74], and L-asparginase has been included in various chemotherapy regimens to treat ANKL patients, such as the SMILE (dexamethasone, methotrexate, ifosfamide, etoposide, and L-asparaginase), AspaMetDex (L-asparaginase, methotrexate, and dexamethasone), or VIDL (etoposide, ifosfamide, dexamethasone, and L-asparaginase) regimens, resulting in improved outcomes [75,76,77], However, no study has compared these regimens in the setting of ANKL [27,76,78]. In addition, one study suggested that the gemcitabine, cisplatin, and dexamethasone (GDP) regimen was efficacious in selected patients [48]. A complete response, including negativity for EBV DNA in the blood after treatment, is associated with a better outcome, including overall survival [76]. However, prognosis remains poor. Patients who achieve a complete remission (CR) after chemotherapy (including L-asparaginase) rarely survive more than one year without further treatment.

### 11.2. Allogeneic Hematopoietic Cell Transplantation in ANKL

Allogeneic hematopoietic stem cell transplantation (SCT) improves outcome in ANKL patients. In a study of eight patients who did not achieve complete remission (CR) before allogeneis SCT, four patients reached CR, and two survived for several years [75]. Subsequent studies also have shown the significant efficacy of allogeneic SCT for ANKL patients [22,76]. A total of 21 ANKL patients enrolled in the International Bone Marrow Transplantation Registry (IBMTR) database underwent allogeneic SCT, with most receiving L-asparaginase-containing chemotherapy before proceeding to transplant [79]. Patients with a CR prior to allogeneic SCT showed a significantly better survival after two years than those without a CR (38 vs. 0%) [79]. This study demonstrated that transplant can provide durable disease control in a subset of ANKL patients who achieve CR before transplantation. However, 76% of all patients died in the long run, mostly due to ANKL [79]. In summary, allogeneic SCT in the setting ANKL might help extend survival in some patients, but success appears rather limited, and novel therapies are needed for ANKL patients.

### 11.3. Novel Therapeutic Applications

Vulnerability of NK cells to targeted therapies could be better assessed through RNA sequencing and drug sensitivity profiling of normal NK cells, as well as cells derived from NK cell neoplasms, opening the gate to the practice of precision medicine in ANKL patients.

#### 11.3.1. BCL2 Inhibitors

Dufva et al. investigated drug sensitivity profiling of nine NK cell lines, including three ANKL and two ENKTL cell lines [62]. They concluded that NK cells show particular sensitivity to inhibition of IL2–JAK–STAT signaling compared with other hematopoietic cells. The authors also reported high efficacy of the *JAK* inhibitor ruxolitinib and the *BCL2* family inhibitor navitoclax across all cell lines (neoplastic and non-neoplastic) [62]. In addition, they observed that the *BCL2* inhibitor venetoclax and mTOR inhibitors were effective only against malignant NK cell lines [62]. Venetoclax was less effective as a single agent compared with the more broad-spectrum BCL2 family inhibitor navitoclax, but venetoclax exerted a synergistic effect with ruxolitinib more consistently and across all cell lines, especially with the addition of the *Aurora kinase (AURK)* inhibitor alisertib [62]. In keeping with these findings, El Hussein et al. demonstrated BCL2 protein overexpression in six of eight ANKL cases, underscoring potential therapeutic applicability of BCL2 inhibitors in clinical settings [19].

#### 11.3.2. Heat Shock Protein 90 (HSP90) Inhibitors

Other effective drug classes reported by Dufva et al. were heat shock protein 90 (HSP90) inhibitors, Polo-like kinase *(PLK)* and cyclin-dependent kinase inhibitors, as well as histone deacetylase inhibitors [62]. On the other hand, most NK cell lines were resistant to MEK inhibitors [62]. Glucocorticoids were highly effective against healthy NK cells compared to other cell types, but induced responses only in few cell lines, implying glucocorticoid resistance in a subset of malignant NK cells [62]. Consistent with the role of *HSP90* in *JAK/STAT* regulation [80], *JAK* and *HSP90* inhibitors as well as the *Nedd8 activating enzyme (NAE)* inhibitor pevonedistat were more effective in IL-2 stimulation than resting NK cells, suggesting that the sensitivity of NK cells to *JAK* and *HSP90* inhibition results largely from inhibition of IL-2-derived *JAK/STAT* activation.

The role of the polycomb repressive complex 2 (PRC2) has been elucidated in the biology of T and NK cell lymphomas [81,82,83,84]. PRC2 is a multiprotein complex composed of several core components including EZH2, a histone methyltransferase responsible for epigenetic silencing of target genes via trimethylation of H3K27. Increased or unchecked PRC2 activity hypermethylates H3K27, leading to repression of tumor suppressor genes [44,85]. In one study, EZH2 and its’ effector H3K27me3 were shown to be overexpressed by IHC in all three EBV-negative ANKL patients [44]. As we have discussed above, activation of the JAK/STAT pathway is suggested to play a major role in overexpression of MYC [63]. MYC, in turn, has been shown to interact with PRC2 through EZH2 and other cofactors such as SUZ12/EED, and is responsible for inducing histone modification of H3K27me3 [86,87,88]. This axis potentially contributes to the pathogenesis of NK cell neoplasms in general, and ANKL in particular. This observation also provides new insights into potential druggable targets in ANKL through the utilization of EZH2 inhibitors.

#### 11.3.3. Other Approaches

Consideration of specific targeted therapies using ex vivo drug screening on patient-derived xenografts might offer another approach for experimental therapies in patient with ANKL. Such an approach has shown promise in patients with T cell neoplasms [89]. One example might be targeting CD38 with daratumumab, as CD38 has already been shown to be expressed in ANKL [12,20].

## 12. Conclusions

Although the outcome of patients with ANKL is poor, and our understanding of ANKL has evolved only slowly over time, substantial recent progress has been made in deciphering the molecular alterations orchestrating the oncogenesis of ANKL. Pharmacologic experiments with therapeutic agents tailored to optimize attack on leukemic NK cells and target recently characterized pathways in ANKL, such as JAK/STAT, hold promise for the future. Implementing the data collected from anecdotal observations into clinical trials, coupled with the creation of a multi-institutional genomic bank of ANKL samples that can be analyzed by high throughput molecular methods, represents steps on the journey towards understanding the biology of and designing effective therapies for this disease.

## Figures and Tables

**Table 1:** An integrative clinicopathologic overview, comparing multiple aspects of NK cell proliferative disorders.

Diagnosis	Etiology	Immunophenotype	Functionality	Genomic Landscape
Transient increase in NK cells	Autoimmune disorders, viral infections			
CLPD*	Unknown stimulus, possibly viral	CD8+ (uniform positivity), CD16+, CD56+ (dim); Loss of CD2, CD7 and CD57	Cytotoxic function	Activating mutations in the STAT3 SH2 domain
ENKTL*	Strong association with EBV infection	CD2+, CD56+; Loss of sCD3, CD4, CD8, CD16 and CD57	Cytokine secretion function	DDX3X, JAK/STAT signaling pathway (STAT3, STAT5B, JAK3, and PTPRK), KIT, CTNNB1, TP53, PRDM1, ATG5, AIM1,FOX03, and HACE1, RAS, MYC, KMT2D/MLL2, ARID1A, EP300, ASXL3, CDKN2A, CDKN2B, CDKN1A, FAS
ANKL*	Strong association with EBV infection	CD2+, CD16+, CD56+; Loss of sCD3, CD4, CD7, CD8 and CD57	Cytokine secretion function	JAK/STAT signaling pathway, TP53, TET2, CREBBP, ASXL1, ASXL2, BRINP3, PRPF40B

*CLPD: chronic lymphoproliferative disorder; *ENKTL: extra-nodal NK/T cell lymphoma; *ANKL: aggressive NK cell leukemia.

**Table 2:** Immunophenotypic characteristics of aggressive NK cell leukemia.

Consistently Positive Markers	Frequently Positive Markers	Frequently Negative Markers
CD2	CD7	CD3
Cytoplasmic CD3 epsilon	CD8	CD4
CD16		CD5
CD56		CD57
CD94		KIR (CD158a–e)
Granzyme B		T-cell receptors
TIA		
Perforin		

**Table 3:** Breakdown of the common mutations reported in the three most recent genomic studies of aggressive NK cell leukemia.

Mutations	Huang et al. [63] (29 Patients)	Dufva et al. [62] (14 Patients)	El Hussein et al. [19] (6 Patients)
JAK/STAT	STAT3, STAT5B, STAT5A, JAK2, JAK3, STAT6, SOCS1, SOCS3 and PTPN11 (48%)	STAT3 (21%)	JAK1, JAK3, STAT3 (66.6%)
RAS/MAPK	--	(29%)	(16.7%)
Epigenetic modifiers	TET2 (28%), CREBBP (21%), KMT2D (21%), BCOR (3%)	SETD2, KMT2D and BCOR (50%), TET2 (7%)	TET2 (16.7%), CREBBP (16.7%), GFI1 (16.7%)
RNA helicase (DDX3X)	(7%)	(21%)	--
Cell cycle regulation and DNA damage repair	TP53 (34%)	TP53 (7%)	TP53 (50%), ASXL1 (33.3%), ASXL2 (33.3%), BRINP3 (16.7%)
mRNA splicing	--	--	PRPF40B (16.7%)
